# The Clinical Significance of Increased Serum Proinflammatory Cytokines, C-Reactive Protein, and Erythrocyte Sedimentation Rate in Patients with Hidradenitis Suppurativa

**DOI:** 10.1155/2017/2450401

**Published:** 2017-07-10

**Authors:** D. Jiménez-Gallo, R. de la Varga-Martínez, L. Ossorio-García, C. Albarrán-Planelles, C. Rodríguez, M. Linares-Barrios

**Affiliations:** ^1^Unidad de Gestión Clínica de Dermatología, Hospital Universitario Puerta del Mar, Cádiz, Spain; ^2^Unidad de Gestión Clínica de Hematología e Inmunología, Hospital Universitario Puerta del Mar, Cádiz, Spain

## Abstract

**Objectives:**

To assess inflammatory serum markers including serum proinflammatory cytokines, C-reactive protein (CRP), and erythrocyte sedimentation rate (ESR) according to the clinical inflammatory activity of patients with hidradenitis suppurativa (HS).

**Patients and Methods:**

Seventy-four patients with HS were studied based on the Hidradenitis Suppurativa-Physician Global Assessment (HS-PGA) score and Hurley staging system. Proinflammatory cytokines were measured using a multiplex cytokine assay. Twenty-two healthy volunteers were recruited.

**Results:**

Serum interleukin- (IL-) 6, IL-23, soluble tumour necrosis factor alpha (TNF-*α*) receptor I (sTNF-RI), CRP, and ESR were different in the patients with HS compared with those in the healthy controls (*P* < 0.05). The levels of IL-1*β*, IL-6, IL-8, IL-10, IL-12p70, IL-17A, sTNF-RII, CRP, and ESR were significantly elevated according to inflammatory activity based on HS-PGA scores (*r* > 0.25, *P* < 0.05). The levels of IL-6 (*r* = 0.53, *P* < 0.001), CRP (*r* = 0.54, *P* < 0.001), and ESR (*r* = 0.60, *P* < 0.001) were especially well correlated with clinical inflammatory activity based on HS-PGA scores. The levels of IL-6, IL-8, sTNF-RI, sTNF-RII, CRP, and ESR were significantly elevated according to Hurley staging system.

**Conclusions:**

Serum proinflammatory cytokines, CRP, and ESR are increased in relation to the clinical inflammatory activity of patients with HS compared with healthy controls. Serum IL-6, CRP, and ESR are effective biomarkers for evaluating the severity of HS.

## 1. Introduction

Hidradenitis suppurativa (HS) is a chronic inflammatory disease originating in the follicular infundibulum [1]. HS is characterized by the formation of multiple inflammatory lesions such as nodules, abscesses, and fistulae, which predominantly affect the intertriginous regions. These lesions are painful and suppurative and result in scar formation in the most severe cases. The most commonly affected areas are the axillae and groin as well as the submammary, gluteal, and perianal regions [2, 3]. The prevalence of HS is estimated to be 1% [2]. Obesity and tobacco use are strongly associated with HS [4, 5]. Pathogenesis of HS is not completely understood. The proposed mechanism of the pathogenesis of HS begins with an alteration of the innate immune response to the commensal microbiota, which leads to epidermal hyperplasia. This epidermal hyperplasia occludes the follicular infundibulum through the formation of cysts, which eventually break, free the keratin fibres, and release the commensal bacteria into the dermis. These steps amplify the inflammatory response through inflammasome activation and the interleukin- (IL-) 1*β*-IL-23/T-helper (Th) 17/IL-17 pathway [3, 6]. Zee et al. [7] studied samples of lesional and perilesional skin from patients with HS and observed elevated levels of IL-1*β*, tumour necrosis factor alpha (TNF-*α*), and IL-10. They also noted that the TNF-*α* levels were 5 times higher in the skin affected by HS than the skin affected by psoriasis. This result is interesting as it implies a greater inflammatory load in HS compared with other dermatological diseases [8, 9]. In this sense, HS continues to be a challenging dermatological disorder for dermatologists [10].

IL-1*β* is a key inflammatory mediator in HS. IL-1*β* levels in skin affected with HS show a trend towards a positive correlation with disease severity [6, 7]. Lima et al. [11] demonstrated the presence of IL-17 in lesional and perilesional skin from patients with HS. IL-17A is the defining cytokine of Th17 lymphocytes [12]. IL-1*β*, IL-6, IL-23, and transforming growth factor-*β* (TGF-*β*) are cytokines that are produced by activated innate cells, and they drive the differentiation of Th17 lymphocytes [6]. IL-17-stimulated keratinocytes secrete IL-1*β* through a mechanism involving the Nod-like receptor protein 3 (NLRP3) inflammasome [13]. Furthermore, IL-10 is an anti-inflammatory cytokine that has also been observed in the skin of patients with HS [6]. Other proinflammatory cytokines such as IL-22, IL-23, IL-6, and IL-8 have shown contradictory results in skin affected with HS [14–16]. In the later phases of HS, infiltration of the dermis by neutrophils leads to the amplification of the inflammatory response with higher production of IL-17 [12]. All of the above provokes an inflammatory loop that is manifested clinically by inflammatory lesions and the suppuration characteristics of HS.

Hessam et al. [17] recently demonstrated that C-reactive protein (CRP), which is an acute phase protein (APP), is effective for evaluating the severity and degree of inflammation in patients with HS. Pascual et al. [18] evaluated subclinical arteriosclerosis in patients with HS and found that the erythrocyte sedimentation rate (ESR) was higher in these patients. The ESR, which is an indirect acute phase reactant, reflects plasma viscosity and the presence of APP, especially fibrinogen [19]. Matusiak et al. [20, 21] described elevated serum levels of chitinase-3-like protein 1 (YKL-40) and IL-17 in patients with HS based on Hurley staging system.

The study of inflammatory markers in immunological diseases with high inflammatory loads, such as rheumatoid arthritis and systemic lupus erythematosus, has resulted in the identification of biomarkers that are used in clinical practice, including serum inflammatory cytokines and CRP [22, 23]. In this study, we analysed the clinical significance of serum proinflammatory cytokines, CRP, and ESR in patients with HS.

## 2. Materials and Methods

### 2.1. Patients and Controls

In total, 74 patients with HS (36 males and 38 females) and a mean ± standard deviation (SD) age of 37.4 ± 12.0 years (range 18–62) were enrolled. HS was diagnosed according to well-established criteria [24]. The exclusion criteria were the following: patients younger than 18 years of age; patients suffering from infectious, tumoural or autoimmune diseases; and patients who were receiving ongoing local or systemic treatment for at least 3 months prior to entering the study. The clinical severity of the disease was evaluated by the same dermatologist using the HS-PGA score (mean ± SD 3.0 ± 0.9) and Hurley staging system. The HS-PGA score is a simple and dynamic scale that allows for the tracking of the clinical progression of inflammation in HS [9, 25]. HS-PGA scores categorize the severity of HS in 6 degrees as a function of the number of noninflammatory nodules, inflammatory nodules, abscesses, and draining fistulae [9, 26, 27]. We divided the patients with HS into the following 3 groups according to clinical inflammatory activity: a group with low inflammatory activity (LIA) (*n* = 22), including patients with HS-PGA scores from 0 to 2 (clear, minimal, and mild); a group with moderate inflammatory activity (MIA) (*n* = 35), including patients with HS-PGA scores of 3 (moderate); and a group with high inflammatory activity (HIA) (*n* = 17), including patients with HS-PGA scores of 4 and 5. Additionally, we divided the patients with HS into the three-degree scale proposed by Hurley [9]. As a control group, 22 healthy volunteers were recruited for this study. This group comprised healthy volunteers without HS who also had no family history of HS. The characteristics of the patients and healthy controls are shown in Table 1. This study was conducted in accordance with the Helsinki Conference (64th World Medical Association General Assembly, Fortaleza, Brasil, October 2013) and with the Spanish legislation on clinical research and personal data protection. The participants' rights and safety took precedence, and approval was obtained from the Hospital Puerta del Mar Ethics Committee. All of the participants were provided detailed information regarding the purpose and method of this study, as well as the expected results, and informed consent was obtained before screening.

### 2.2. Serum

Venous blood samples (5–10 mL) were collected from the patients and healthy controls into vacuum tubes under sterile conditions. The serum was separated by centrifugation while the samples were fresh, immediately frozen at −80°C, and then stored until processing.

### 2.3. Cytokine Assays

Multiple cytokine analyses were performed using xMAP technology (Luminex Corporation, Austin, TX, USA) to measure the serum levels of IL-1*β*, IL-6, IL-8, IL-10, IL-12p70, IL-17A, IL-22, IL-23, and soluble TNF receptors I (sTNF-RI) and II (sTNF-RII). The Milliplex MAP multiplex assay was conducted in a 96-well microplate according to the manufacturer's recommendations (Millipore, Billerica, MA, USA). The levels of the serum inflammatory cytokines were calculated using a standard curve, and the minimum detectable concentrations were 0.18 pg/mL for IL-1*β*, 0.23 pg/mL for IL-6, 0.79 pg/mL for IL-8, 0.94 pg/mL for IL-10, 0.33 pg/mL for IL-12p70, 0.58 pg/mL for IL-17A, 0.04 pg/mL for IL-22, 0.12 pg/mL for IL-23, 12.2 pg/mL for sTNF-RI, and 12.2 pg/mL for sTNF-RII. Due to the short half-life of TNF-*α*, the sTNF-R (sTNF-RI, 55 kDa, and sTNF-RII, 75 kDa) levels were quantified because their half-lives are longer. The serum concentrations of the sTNF-Rs correlate with those of TNF-*α* [28]. All of the samples were analysed simultaneously at the end of recruitment, and the researchers were blinded to the clinical data.

### 2.4. Measurement of C-Reactive Protein Levels and the Erythrocyte Sedimentation Rate

The CRP serum levels were measured using a turbidimetric assay with the Cobas 8000 (Roche Diagnostics, Mannheim, Germany). The Westergren method involving the collection of 2 mL of venous blood into a tube containing 0.5 mL of sodium citrate was used to measure the ESR (VES-MATIC 60, Menarini). A CRP level of greater than 6 mg/L and an ESR of greater than 20 mm/hour were regarded as abnormal values.

### 2.5. Statistical Analysis

All data were analysed using SPSS 19.0 for Windows (SPSS, Chicago, IL, USA). All data are presented as the mean ± SD (median). A two-tailed test was used. We used the Mann–Whitney *U* test to evaluate the differences between 2 groups (healthy controls versus patients with HS). We then divided the patients into 3 groups according to clinical inflammatory activity based on their HS-PGA scores. The Kruskal-Wallis test was used to make comparisons among the healthy controls, patients with LIA HS, patients with MIA HS, and patients with HIA HS. To examine whether there were any statistically significant differences among the 4 groups, Bonferroni's post hoc test for pairwise comparisons was used. The correlation analysis was performed by calculating the Spearman's correlation coefficient. Thus, the effect size was small (0.10–0.29), medium (0.30–0.49), and large (above 0.50), as defined by Cohen [29]. Additionally, the Kruskal-Wallis test was used to make comparisons among the healthy controls and patients with HS based on Hurley staging system. Statistical significance was accepted as *P* < 0.05.

## 3. Results

### 3.1. A Comparison of the Levels of Inflammatory Serum Markers between Patients with Hidradenitis Suppurativa and the Control Group

The serum levels of 10 cytokines and CRP, as well as the ESR, were analysed in 74 patients with HS and 22 healthy controls (Table 2). The levels of IL-6, IL-23, sTNF-RI, and CRP, as well as the ESR, were significantly different in our experimental group (*P* < 0.05). These findings are interesting because they reveal the systemic inflammatory characteristics of the patients with HS.

### 3.2. A Comparison of the Levels of Inflammatory Serum Markers among the Healthy Control, Low Inflammatory Activity, Moderate Inflammatory Activity, and High Inflammatory Activity Groups

**Figure 1:**
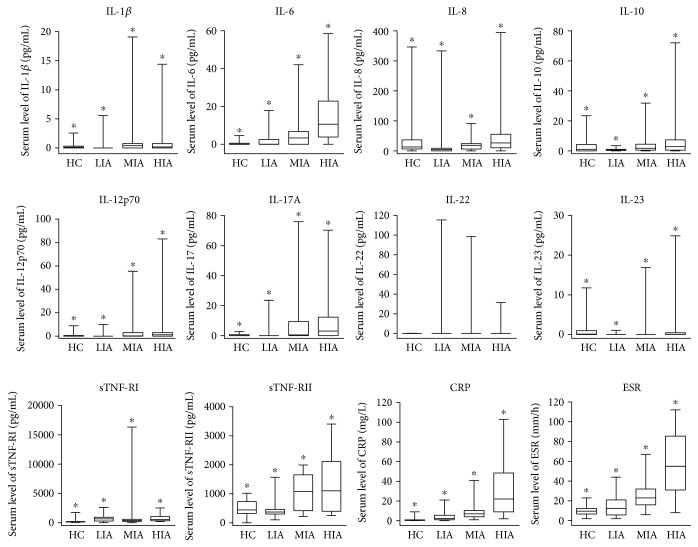
Comparison of inflammatory serum marker levels among healthy controls and patients with hidradenitis suppurativa according to clinical inflammatory activity. IL, interleukin; CRP, sTNF-RI, soluble TNF receptor I; sTNF-RII, soluble TNF receptor II; C-reactive protein; ESR, level erythrocyte sedimentation rate; HC, healthy controls; LIA, low inflammatory activity; MIA, moderate inflammatory activity; HIA, high inflammatory activity.

We divided the patients with HS into the following 3 groups according to clinical inflammatory activity based on their HS-PGA scores: a LIA HS group (*n* = 22) including patients with HS-PGA scores from 0 to 2 (clear, minimal, and mild); a MIA HS group (*n* = 35) including patients with HS-PGA scores of 3 (moderate); and a HIA HS group (*n* = 17) including patients with HS-PGA scores of 4 and 5 (severe and very severe, resp.) (Table 3). When comparing the 4 groups (3 groups of HS patients and 1 control group), statistically significant differences in the levels of IL-1*β*, IL-6, IL-8, IL-10, IL-12p70, IL-17A, IL-23, sTNF-RI, sTNF-RII, and CRP, as well as the ESR, were observed (Figure 1). The cytokines that were significantly different when comparing the 4 groups were then analysed using Bonferroni's post hoc test for pairwise comparisons (Table 4). Interestingly, there were no significant differences in the levels of inflammatory serum markers between the healthy control group and the LIA HS group. Additionally, sTNF-RII levels and ESR were the most precocious inflammatory markers, as they showed the earliest changes with increasing severity of HS. Finally, the changes in IL-6 and CRP levels, as well as the ESR, were significant in the MIA HS group and the HIA HS group, highlighting the progression of inflammation in the groups with higher inflammatory loads.

### 3.3. Correlations between Inflammatory Serum Markers and Disease Severity

The serum levels of the 10 cytokines, as well as the CRP levels and the ESR, in the 74 patients with HS were measured and aligned with disease severity (based on clinical inflammatory activity categorized according to HS-PGA scores). The levels of IL-1*β*, IL-6, IL-8, IL-10, IL-12p70, IL17A, sTNF-RII, and CRP, as well as the ESR, showed significant linear correlations with clinical inflammatory activity in the patients with HS (Table 5). However, only IL-6, CRP, and ESR had absolute *r* values that were greater than 0.49, indicating a strong association. These last 3 may be potential biomarkers for HS.

### 3.4. A Comparison of the Levels of Inflammatory Serum Markers among the Healthy Control and the Three-Degree Scale Proposed by Hurley

The serum levels of the 10 cytokines, as well as CRP levels and the ESR, in the 74 patients with HS were measured and aligned with disease severity (based on clinical inflammatory activity categorized according to Hurley staging system). The levels of IL-6, IL-8, sTNF-RI, sTNF-RII, CRP, and ESR were significantly elevated according to Hurley staging system (Table 6). The loss of statistical significance for IL-17A could be due to greater difficulty in categorizing clinical inflammation for Hurley staging.

## 4. Discussion

The valuation of the severity of an inflammatory disease requires a correlation between clinical inflammatory activity and laboratory parameters [22, 23]. Various validated tools are available to evaluate the clinical severity of HS. The Hurley staging system is a simple but static scale that does not measure the number of inflammatory lesions in each area [9]. The modified Sartorius score combines global and local measurements and is widely used, but because of its hybrid nature, the HS-PGA score is also currently used. The HS-PGA score focuses on inflammatory lesions and is perfectly suitable for the daily assessment of pharmaceutical treatments [25, 27]. Studies of inflammatory serum markers in HS are scarce. Blok et al. [30] studied the evolution of serum levels of IL-2R, TNF-*α*, IL17A, and IL17F in 12 patients who were treated with ustekinumab, a monoclonal antibody against IL-12/23 p40, and they did not observe an elevation of these inflammatory markers or changes with treatment. Matusiak et al. [31] studied the serum TNF-*α* levels of 54 patients with HS and observed significantly higher levels in the HS patients compared with the healthy controls. In another study, they also identified the soluble IL-2 receptor as a potential marker of value for determining the severity of HS [32]. These articles initiated the study of serum inflammatory cytokines as biomarkers for HS. In the present study, we measured the serum levels of 10 proinflammatory cytokines (IL-1*β*, IL-6, IL-8, IL-10, IL-12p70, IL-17A, IL-22, IL-23, sTNF-RI, and sTNF-RII), as well as CRP levels and the ESR, in patients with HS and healthy controls. Additionally, we analysed them according to the level of inflammatory activity (categorized according to HS-PGA scores and Hurley staging system). As we expected, the levels of proinflammatory cytokines, as well as CRP levels and the ESR, were elevated in the patients with HS compared with the healthy controls. The levels of IL-1*β*, IL-6, IL-8, IL-10, IL-12p70, IL17A, sTNF-RI, and CRP, as well as the ESR, showed a significant linear correlation with clinical inflammatory activity in the patients with HS based on their HS-PGA scores. In this sense, our results are consistent with the activation of the IL-1*β*-IL-23/Th17/IL-17 pathway in patients with HS [1, 3, 6], but they reflect systemic expression. These results show that these inflammatory markers differ according to the clinical inflammatory activity of HS. Additionally, there were no significant differences between the healthy control group and the LIA HS group. Thus, there may be different inflammatory phenotypes of HS. Regarding Hurley staging system, this scale could be more difficult to categorize systemic inflammation in HS.

The treatment of HS is challenging for dermatologists. Adalimumab is a TNF-*α* blocker that is the only approved agent for the treatment of moderate to severe HS in the EU and the USA. Infliximab is another TNF-*α* blocker that is also used to treat HS, which is supported by strong evidence [33, 34]. Other biologics such as anakinra, an IL-1 receptor antagonist, and ustekinumab have been explored, but more data are necessary in order to assign them a role in the treatment of HS [34]. In this context, inflammatory serum markers could be used to identify those individuals who would benefit from early treatment to reduce exposure to potential side effects and the high costs of biologic therapy. Furthermore, the concept of a “window of opportunity” is sensible, as it can allow for the identification of patients with high systemic inflammatory HS loads in the early stages to prevent complications such as scarring and complex fistulae and halt the inflammatory progression of the disease. From this point of view, we would like to emphasize that in our study, the sTNF-RII levels and ESR were the most precocious inflammatory markers, as they showed early changes with the increased severity of HS. Thus, these may be suitable for use as early biomarkers to detect the start of systemic inflammation in HS. In a study by Hessam et al., significantly positive correlations of neutrophil count (*r* = 0.330) and CRP levels (*r* = 0.496) with clinical inflammatory activity in accordance with the modified Hidradenitis Suppurativa score were demonstrated [17]. These results are in line with those of our study, with the most important results centering on the strength of the associations for IL-6 (*r* = 0.53), CRP (*r* = 0.54), and ESR (*r* = 0.60), which were greater than 0.5 and similar to those described in the literature for CRP [17].

The role of IL-6 in the pathogenesis of HS is unclear, as IL-6 levels in the skin affected by HS are variable [16]. As we have mentioned previously, IL-6 along with IL-23 and IL-1*β* drive the development of Th17 lymphocytes [6, 35]. Additionally, IL-6 is secreted by neutrophils and macrophages during inflammatory conditions, such as during stimulation of Toll-like receptor- (TLR-) 4 by lipopolysaccharide or stimulation of cells by IL-1 or TNF-*α* [36]. Furthermore, IL-6 induces the expression of APPs such as CRP [16, 17]. For this reason, CRP and IL-6 levels in our study showed correlations with clinical inflammatory activity that were practically equal. The role of neutrophils in the later phase of the pathogenesis of HS has been demonstrated [12], and this neutrophilic activation was determined based on increased neutrophil counts and CRP levels in laboratory tests [17]. Thus, we hypothesize that the elevation of serum IL-6 and CRP levels is the final result of intense neutrophilic activation in the most severe forms of HS. In short, IL-6 probably does not initially activate the IL-1*β*-IL-23/Th17/IL-17 pathway in the skin affected by HS. Circulating IL-6 would be secreted into the serum after the triggering of neutrophil activation by Th17 lymphocytes. This circulating cytokine may initiate the final step of systemic inflammation in HS by liberating CRP in the liver. HS may be an autoinflammatory dermatological disorder due to its initiation of inflammatory responses through IL-1*β* and inflammasomes, or it could be considered a neutrophilic disorder due to the increased activation of neutrophils, which is reflected by circulating IL-6 levels.

## 5. Conclusion

We found that patients with HS have higher levels of serum proinflammatory cytokines, CRP, and ESR compared with healthy controls. The levels of serum proinflammatory cytokines vary according to the degree of clinical inflammation. These inflammatory serum markers reflect the systemic inflammatory load of patients with more severe forms of HS. Serum levels of the inflammatory markers IL-6 and CRP, as well as the ESR, are effective for evaluating the severity and degree of inflammation in patients with HS.

## Figures and Tables

**Table 1:** Demographics of healthy controls and patients with hidradenitis suppurativa.

	Healthy controls	Patients with hidradenitis suppurativa
*n*	22 (10 males, 12 females)	74 (36 males, 38 females)
*Age (years), mean ± SD*	37.4 ± 13.4	37.4 ± 12.0
*Smoking, current smoker, n (%)*	0 (0%)	57 (77)
*BMI, kg/m^2^*		
Normal weight: BMI ≤ 24.9, *n* (%)	Normal weight: 20 (91.0)	Normal weight: 18 (24.3)
Overweight: BMI ≥ 25, *n* (%)	Overweight: 2 (9.1)	Overweight: 24 (32.4)
Obesity: BMI ≥ 30, *n* (%)		Obesity: 32 (43.2)
*HS-PGA, mean ± SD*		3.0 ± 0.9
*Clinical inflammatory activity*		
LIA: HS-PGA ≤ 2, *n* (%)		LIA: 22 (29.7)
MIA: HS-PGA = 3, *n* (%)		MIA: 35 (47.3)
HIA: HS-PGA ≥ 4, *n* (%)		HIA: 17 (23.0)
*Hurley staging system*		Stage I: 11 (14.9)
		Stage II: 47 (63.5)
		Stage III: 16 (21.6)

BMI: body mass index; HS-PGA: Hidradenitis Suppurativa-Physician Global Assessment; LIA: low inflammatory activity; MIA: moderate inflammatory activity; HIA: high inflammatory activity. There were no significant differences in age and male/female ratio between the patients with hidradenitis suppurativa and healthy controls (*P* > 0.05).

**Table 2:** Comparison of inflammatory serum marker levels among healthy controls and patients with hidradenitis suppurativa, and the results of statistical assessments.

Cytokine levels (pg mL^−1^); CRP (mg L^−1^); ESR (mm hour^−1^), mean ± SD (median)			
	Healthy controls	Hidradenitis suppurativa	*P* value^a^
IL-1*β*	0.4 ± 0.8 (0.13)	0.9 ± 2.9 (0.00)	0.801
IL-6	0.6 ± 1.3 (0.00)	6.2 ± 10.7 (3.00)	0.001^∗^
IL-8	36.3 ± 73.9 (12.60)	27.9 ± 62.3 (13.62)	0.442
IL-10	3.3 ± 5.8 (0.97)	3.4 ± 9.2 (0.64)	0.979
IL-12p70	0.6 ± 1.9 (0.01)	3.4 ± 12.1 (0.00)	0.427
IL-17A	0.3 ± 0.6 (0.00)	5.6 ± 12.1 (0.00)	0.147
IL-22	0.0 ± 0.1 (0.00)	8.8 ± 25.6 (0.00)	0.950
IL-23	1.0 ± 2.5 (0.09)	0.9 ± 3.6 (0.00)	0.014^∗^
sTNF-RI	325.9 ± 507.1 (145.81)	879.8 ± 1914.7 (504.39)	<0.001^∗^
sTNF-RII	527.4 ± 266.9 (438.65)	927.9 ± 722.6 (507.19)	0.053
CRP	1.2 ± 2.1 (0.55)	13.4 ± 19.2 (7.00)	<0.001^∗^
ESR	10.2 ± 5.5 (9.50)	29.5 ± 23.9 (23.00)	<0.001^∗^

IL: interleukin; sTNF-RI: soluble TNF receptor I; sTNF-RII: soluble TNF receptor II; CRP: C-reactive protein; ESR: level erythrocyte sedimentation rate. ^a^Mann–Whitney *U* test between healthy controls and patients with hidradenitis suppurativa. ^∗^*P* < 0.05.

**Table 3:** Comparison of inflammatory serum marker levels among healthy controls and patients with hidradenitis suppurativa according to clinical inflammatory activity based on their HS-PGA scores.

Cytokine levels (pg mL^−1^); CRP (mg L^−1^); ESR (mm hour^−1^), mean ± SD (median)					
	Healthy controls	Low inflammatory activity	Moderate inflammatory activity	High inflammatory activity	*P* value^a^
IL-1*β*	0.4 ± 0.8 (0.13)	0.4 ± 1.4 (0.00)	1.1 ± 3.2 (0.39)	1.3 ± 3.5 (0.19)	0.022^∗^
IL-6	0.6 ± 1.3 (0.00)	1.8 ± 3.9 (0.00)	4.6 ± 7.2 (3.38)	15.4 ± 16.3 (10.65)	<0.001^∗^
IL-8	36.3 ± 73.9 (12.60)	21.7 ± 70.4 (4.13)	17.6 ± 16.2 (17.89)	57.1 ± 97.3 (26.24)	0.003^∗^
IL-10	3.3 ± 5.8 (0.97)	0.7 ± 0.7 (0.46)	3.2 ± 5.7 (1.63)	7.5 ± 17.0 (2.82)	0.032^∗^
IL-12p70	0.6 ± 1.9 (0.01)	0.6 ± 2.2 (0.00)	4.2 ± 10.8 (0.15)	6.6 ± 19.9 (1.20)	0.022^∗^
IL-17A	0.3 ± 0.6 (0.00)	1.4 ± 5.2 (0.00)	6.4 ± 13.6 (0.40)	9.1 ± 16.8 (2.97)	0.001^∗^
IL-22	0.0 ± 0.1 (0.00)	12.9 ± 33.7 (0.00)	9.5 ± 25.3 (0.00)	1.9 ± 7.6 (0.00)	0.904
IL-23	1.0 ± 2.5 (0.09)	0.1 ± 0.3 (0.00)	0.9 ± 3.2 (0.00)	1.7 ± 6.0 (0.00)	0.026^∗^
sTNF-RI	325.9 ± 507.1 (145.81)	746.0 ± 634.2 (645.27)	1000.3 ± 2714.8 (390.42)	804.8 ± 689.2 (577.87)	<0.001^∗^
sTNF-RII	527.4 ± 266.9 (438.65)	491.4 ± 380.2 (368.32)	1017.1 ± 583.3 (1077.75)	1309.0 ± 1017.6 (1096.81)	<0.001^∗^
CRP	1.2 ± 2.1 (0.55)	4.8 ± 5.8 (2.25)	9.6 ± 9.8 (7.00)	32.2 ± 30.5 (22.00)	<0.001^∗^
ESR	10.2 ± 5.5 (9.50)	15.3 ± 12.3 (12.50)	25.2 ± 13.2 (23.00)	56.7 ± 30.6 (55.00)	<0.001^∗^

HS-PGA: Hidradenitis Suppurativa-Physician Global Assessment; IL: interleukin; CRP: C-reactive protein; sTNF-RI: soluble TNF receptor I; sTNF-RII: soluble TNF receptor II; ESR: level erythrocyte sedimentation rate. ^a^Kruskal-Wallis test comparing the circulating levels of inflammatory cytokines among the healthy controls and patients with hidradenitis suppurativa according to clinical inflammatory activity. ^∗^*P* < 0.05.

**Table 4:** Bonferroni's post hoc test for pairwise comparisons among the four groups based on their HS-PGA scores.

Group	IL-6	sTNF-RII	CRP	ESR
Healthy controls versus low inflammatory activity				
Healthy controls versus moderate inflammatory activity		^∗^		^∗^
Healthy controls versus high inflammatory activity	^∗^	^∗^	^∗^	^∗^
Low inflammatory activity versus moderate inflammatory activity		^∗^		
Low inflammatory activity versus high inflammatory activity	^∗^	^∗^	^∗^	^∗^
Moderate inflammatory activity versus high inflammatory activity	^∗^		^∗^	^∗^

HS-PGA: Hidradenitis Suppurativa-Physician Global Assessment; IL: interleukin; sTNF-RII: soluble TNF receptor II; CRP: C-reactive protein; ESR: level erythrocyte sedimentation rate. ^∗^*P* < 0.05.

**Table 5:** Correlation among clinical inflammatory activity and inflammatory serum marker levels in patients with hidradenitis suppurativa based on their HS-PGA scores.

Statistical values		
	*r*	*P* value
IL-1*β*	0.28	0.016^∗^
IL-6	0.53	<0.001^∗^
IL-8	0.41	<0.001^∗^
IL-10	0.34	0.003^∗^
IL-12p70	0.30	0.008^∗^
IL-17A	0.37	0.001^∗^
IL-22	0.02	0.874
IL-23	0.23	0.05
sTNF-RI	0.00	0.998
sTNF-RII	0.4	<0.001^∗^
CRP	0.54	<0.001^∗^
ESR	0.60	<0.001^∗^

HS-PGA: Hidradenitis Suppurativa-Physician Global Assessment; IL: interleukin; CRP: C-reactive protein; sTNF-RI: soluble TNF receptor I; sTNF-RII: soluble TNF receptor II; ESR: level erythrocyte sedimentation rate; *r*: Spearman correlation coefficient. ^∗^*P* < 0.05.

**Table 6:** Comparison of inflammatory serum marker levels among healthy controls and patients with hidradenitis suppurativa according to Hurley staging system.

Cytokine levels (pg mL^−1^); CRP (mg L^−1^); ESR (mm hour^−1^), mean ± SD (median)					
	Healthy controls	Stage I	Stage II	Stage III	*P* value^a^
IL-1*β*	0.4 ± 0.8 (0.13)	0.6 ± 1.7 (0.00)	1.2 ± 3.5 (0.00)	0.4 ± 0.7 (0.09)	0.489
IL-6	0.6 ± 1.3 (0.00)	2.5 ± 5.2 (0.00)	3.3 ± 4.5 (2.07)	17.3 ± 17.5 (10.65)	<0.001^∗^
IL-8	36.3 ± 73.9 (12.60)	8.8 ± 12.9 (6.20)	23.2 ± 52.8 (14.02)	54.8 ± 95.5 (27.97)	0.023^∗^
IL-10	3.3 ± 5.8 (0.97)	0.5 ± 0.4 (0.41)	3.6 ± 10.6 (0.76)	5.0 ± 7.9 (2.05)	0.157
IL-12p70	0.6 ± 1.9 (0.01)	0.3 ± 0.7 (0.00)	5.1 ± 15.0 (0.00)	1.7 ± 2.5 (0.68)	0.281
IL-17A	0.3 ± 0.6 (0.00)	0.7 ± 2.2 (0.00)	6.9 ± 15.5 (0.00)	5.1 ± 6.1 (1.79)	0.073
IL-22	0.0 ± 0.1 (0.00)	0.0 ± 0.0 (0.00)	11.2 ± 28.9 (0.00)	7.6 ± 23.1 (0.00)	0.360
IL-23	1.0 ± 2.5 (0.09)	0.1 ± 0.3 (0.00)	1.2 ± 4.5 (0.00)	0.3 ± 0.6 (0.00)	0.071
sTNF-RI	325.9 ± 507.1 (145.81)	649.9 ± 360.9 (724.17)	595.1 ± 599.2 (401.47)	1874.2 ± 3912.4 (641.13)	<0.001^∗^
sTNF-RII	527.4 ± 266.9 (438.65)	412.3 ± 322.4 (353.40)	947.2 ± 610.2 (814.01)	1225.5 ± 1019.4 (890.28)	0.001^∗^
CRP	1.2 ± 2.1 (0.55)	5.6 ± 7.2 (2.00)	8.3 ± 9.2 (5.00)	33.6 ± 30.8 (22.50)	<0.001^∗^
ESR	10.2 ± 5.5 (9.50)	17.9 ± 12.9 (19.00)	20.8 ± 13.0 (21.00)	62.9 ± 25.0 (55.50)	<0.001^∗^

IL: interleukin; CRP: C-reactive protein; sTNF-RI: soluble TNF receptor I; sTNF-RII: soluble TNF receptor II; ESR: level erythrocyte sedimentation rate. ^a^Kruskal-Wallis test comparing the circulating levels of inflammatory cytokines among the healthy controls and patients with hidradenitis suppurativa according to Hurley staging system. ^∗^*P* < 0.05.
